# Monkeypox and fecal microbiota for transplantation(FMT): An unprecedented risk?

**DOI:** 10.1016/j.amsu.2022.104779

**Published:** 2022-09-24

**Authors:** Rabia Owais, Maham Iqbal

**Affiliations:** Dow University of Health Sciences, Karachi, Pakistan

**Keywords:** Monkeypox virus, Fecal microbiota for transplantation

## 1 Introduction

The monkeypox virus (MPVX) a double-stranded DNA virus, belongs to the orthopoxvirus genus of the poxviridae family [1]. Since 1970, 11 African countries have reported human cases of the monkeypox virus [1,2]. The observed mortality rate for nonimmunized individuals is between 0% and 11%. An outbreak of monkeypox was confirmed in the United Kingdom on May 6, 2022. By May 21, 2022, 92 cases were confirmed worldwide, from 13 countries where the monkeypox virus (MPXV) was not endemic [2].

Microbiota refers to the range of microbial species that live inside the human body and interact with it [3]. The gastrointestinal tract resides in over 98% of the gut microbiota [3]. The transfer of intestinal bacteria from a healthy donor into a recipient to alter the recipient's intestinal microbiome is known as fecal microbiota transplantation (FMT) [1]. The Fecal Microbiota Transplantation (FMT) method is employed to treat illness symptoms and restore dysbiosis. Dysbiosis is an “imbalance” in the gut microbial flora associated with the disease. Data suggests that several pathologies are linked to gut microbial dysbiosis [3]. According to the number of clinical trials, fecal microbiota transplantation may have therapeutic potential for diseases like cancer, inflammatory, infectious, autoimmune, obesity, and metabolic syndrome [3].

## 2 Transmission

One of the largest animal reservoirs for the virus is rodents [3]. They are multiple modes of transmission through which MPVX penetrates through the human body and are hypothesized to further increase its spread. Human MPXV transmission occurs in two ways: from animal to human or human to human [2]. Animal-to-human transmission occurs through a bite, scratch, bush meat preparation, or direct or indirect contact with lesion materials or body fluids [1]. Transmission from human to human occurs through large respiratory droplets e.g., sneezing or coughing or direct contact with body fluids [1]. Transmission through sexual routes is suspected in the patient with MPXV genital lesions [2].

The monkeypox virus can incubate for five to three weeks, and symptoms can appear between two and five weeks after incubation [2]. Fever, chills, headache, muscle pains, backaches, and lethargy are the initial symptoms of MPVX infection, which eventually proceeds to exhaustion [1]. Furthermore, a hallmark clinical manifestation that helps in the differential diagnosis of MPVX is the presence of enlarged lymph nodes, which can be detected in the neck, groin, and submandibular regions, in 90% of patients [2].

Fecal transplant therapies have raised concerns amongst the Food and Drug Administration (FDA) that they may expose patients to monkeypox. A recent study has found monkeypox viral DNA in rectal swabs and feces samples from infected people [4]. Moreover, another investigation by Baetselier et al. confirmed its exposure in three asymptomatic men from rectal swabs and extracted viable viral DNA from two of those swabs, providing evidence that monkeypox virus DNA has been identified in both stool samples and rectal swabs [5]. The infection may be spread by FMT products, parallel to findings in a recent study, however, the likelihood of these occurrences cannot be confirmed yet [4]. In several trials and reviews published, some minor adverse consequences have been demonstrated such as temporary abdominal pain, diarrhea, and moderate pyrexia, were reported after FMT, and rare drastic adverse effects like pneumonia, inflammatory bowel disease, infection, sepsis, and post-infectious irritable bowel syndrome were frequently linked to potential endoscopy and sedation complications [6]. Thus, for any exploratory use of FMT, the FDA has determined that additional safeguards are required due to the possibility of significant side effects [4].

## 3 The pathophysiology

MPVX can enter the body via the oropharynx, nasopharynx, or intradermal pathways [1]. The monkeypox virus replicates at the inoculation site after viral entrance; in human-to-human transmission, the inoculation site is the respiratory and oropharyngeal mucosa. In primary viremia, the viral load multiplies and then spreads to the adjacent lymph nodes. The entire procedure reflects the incubation phase, which can normally last seven to fourteen days [7]. Monkeypox symptoms and clinical manifestations can be correlated to the prodromal stage. Secondary viremia spreads from lymphoid organs to the epidermis and tertiary organs such as the lungs, eyes, gastrointestinal tract, and so on during the prodromal stage. An individual is the most infectious during this stage. This is primarily due to the predominance of non-specific symptoms like mucocutaneous lesions [7]. The FMT procedure poses a considerable risk of infection during this time. Rare procedural, infectious, and inflammatory problems are also connected to FMT. Recently, DeFilipp et al. reported two cases of drug-resistant Escherichia coli bacteremia transmitted by FMT, further supporting the idea that if the donor's microbiota is contaminated with transmissible pathogens, it can pass the recipient's epithelium and cause viral, digestive, and bloodstream infection [8].

## 4 Suggestions

Since the number of cases of monkeypox virus has been drastically amplifying, several studies have found monkeypox virus DNA in rectal swabs and identified gastrointestinal symptoms in infected subjects [5,9] raising serious concerns about possible transmission of the monkeypox virus via the fecal-oral route [4]. Monkeypox transmission through stool samples is still a subject of some debate since there is still a lack of substantial evidence to support these theories therefore more trials are needed to ascertain the link between the virus transmission and FMT treatment.

As FMT is potentially therapeutic in a wide range of conditions, including but not limited to refractory Clostridioides difficile infection, inflammatory bowel diseases, HIV infection, etc., [3]. It is necessary to assess whether the benefits overrule the associated adverse effects associated with the treatment when considering management options for patients in dire need of the procedure. Clinical trials should be conducted to reaffirm the efficacy and safety of this procedure as minor adverse effects were often overlooked in small investigations.

In addition, the donor-screening process should be updated in the context of these recent transmission incidents and attempts should be made to screen for emerging infectious diseases including SARS-CoV-2 and the monkeypox virus.

In conclusion, additional clinical studies are necessary to validate the relationship between monkeypox and FMT and a highly efficacious and reliable test should be designed to detect monkeypox viral DNA in fecal matter.

## Ethical approval

No ethical approval was required for this paper.

## Sources of funding

No funding was acquired for this paper.

## Consent

No consent was required for this paper.

## Informed consent

This study did not involve any volunteers or patients; hence no consent was needed.

## Author contribution

Rabia Owais: Concept of the study, writing the paper, and final approval of the manuscript. Maham Iqbal: writing the paper and final approval of the manuscript.

## Registration of research studies

1. Name of the registry: Not applicable
2. Unique Identifying number or registration ID: Not applicable
3. Hyperlink to your specific registration (must be publicly accessible and will be checked): Not applicable

## Guarantor

Rabia Owais, Maham Iqbal.

## Declaration of competing interest

None.
